# The Dynamics of Genome-wide DNA Methylation Reprogramming in Mouse Primordial Germ Cells

**DOI:** 10.1016/j.molcel.2012.11.001

**Published:** 2012-12-28

**Authors:** Stefanie Seisenberger, Simon Andrews, Felix Krueger, Julia Arand, Jörn Walter, Fátima Santos, Christian Popp, Bernard Thienpont, Wendy Dean, Wolf Reik

**Affiliations:** 1 Epigenetics Programme, The Babraham Institute, Cambridge CB22 3AT, UK; 2 Bioinformatics Group, The Babraham Institute, Cambridge CB22 3AT, UK; 3 Department of Biological Sciences, Institute of Genetics/Epigenetics, University of Saarland, Campus Saarbrücken, 66123 Saarbrücken, Germany; 4 Laboratory of Translational Genetics, Vesalius Research Center, VIB and KULeuven, 3000 Leuven, Belgium; 5 Centre for Trophoblast Research, University of Cambridge, Cambridge CB2 3EG, UK; 6 Wellcome Trust Sanger Institute, Cambridge CB10 1SA, UK

## Abstract

Genome-wide DNA methylation reprogramming occurs in mouse primordial germ cells (PGCs) and preimplantation embryos, but the precise dynamics and biological outcomes are largely unknown. We have carried out whole-genome bisulfite sequencing (BS-Seq) and RNA-Seq across key stages from E6.5 epiblast to E16.5 PGCs. Global loss of methylation takes place during PGC expansion and migration with evidence for passive demethylation, but sequences that carry long-term epigenetic memory (imprints, CpG islands on the X chromosome, germline-specific genes) only become demethylated upon entry of PGCs into the gonads. The transcriptional profile of PGCs is tightly controlled despite global hypomethylation, with transient expression of the pluripotency network, suggesting that reprogramming and pluripotency are inextricably linked. Our results provide a framework for the understanding of the epigenetic ground state of pluripotency in the germline.

## Highlights

► DNA demethylation in PGCs occurs in two phases ► Global loss of methylation reveals evidence for a passive demethylation mechanism ► Global methylation erasure coincides with expression of the pluripotency network ► VECs (variably erased CGIs) may act as carriers of transgenerational inheritance

## Introduction

Epigenetic information in the mammalian genome is relatively stable in differentiated cells of the soma but is reprogrammed on a genome-wide scale in primordial germ cells (PGCs) and early embryos (Reik et al., 2001; Surani et al., 2007; Sasaki and Matsui, 2008; Smith et al., 2012). This includes the erasure of DNA methylation and the large-scale reprogramming of histone modifications and histone variants (Hajkova et al., 2002, 2008; Lee et al., 2002; Lane et al., 2003; Yamazaki et al., 2003; Seki et al., 2005, 2007; Popp et al., 2010; Guibert et al., 2012). A recent interesting insight into reprogramming of histone modifications in PGCs was provided when it was shown that the H3K27me3 demethylase Utx is responsible, at least in part, for the erasure of H3K27me3 in PGCs and the transcriptional activation of some pluripotency genes (Mansour et al., 2012).

PGCs are first formed as a small cluster (around 40 cells) of *Prdm1*-expressing cells in the proximal epiblast at around E7.25, and their specification and further fate are dependent on the transcriptional regulators *Prdm1*, *Prdm14*, and *Tcfap2c* (Magnúsdóttir et al., 2012). These transcriptional regulators appear to be important for the suppression of somatic cell fate in PGCs, and *Prdm14* is at least in part responsible for the induction of epigenetic reprogramming (Yamaji et al., 2008). Early PGCs also express *Nanog*, *Oct4*, and *Sox2*, and pluripotent stem cells (embryonic germ cells, EGCs) can be derived from them (Surani et al., 2007).

Blastocyst-stage embryos including the inner cell mass are globally hypomethylated, as a result of epigenetic reprogramming during preimplantation development, but upon implantation rapid de novo methylation occurs primarily in the epiblast (Howlett and Reik, 1991; Santos et al., 2002; Borgel et al., 2010; Smith et al., 2012). Recent work indicates that early PGCs express de novo methyltransferases just as other epiblast cells do (Kurimoto et al., 2008) and show evidence of high levels of methylation at E8.0–E8.5 by immunofluorescence (IF) with a 5-methylcytosine (5mC) antibody or by bisulfite sequencing of some candidate loci (Seki et al., 2005; Guibert et al., 2012). Based on a reduction of the IF signal after E8.5, it was proposed that genome-wide loss of methylation occurs relatively early, during the migration phase, in PGC development (Seki et al., 2005). Other studies, however, have shown that many individual sequences analyzed by bisulfite sequencing, including some differentially methylated regions (DMRs) in imprinted genes, were demethylated relatively late once PGCs were colonizing the gonads (Hajkova et al., 2002; Lee et al., 2002; Maatouk et al., 2006; Guibert et al., 2012; Hackett et al., 2012a). It is thus unclear how the dynamics of demethylation are orchestrated across the whole genome and potentially across different stages of PGC development.

Knowledge of the mechanisms of demethylation during PGC development is also still in its infancy. The de novo methyltransferases *Dnmt3a* and *Dnmt3b* as well as *Np95* (also known as *Uhrf1*), which targets Dnmt1 to the DNA replication machinery to maintain methylation during mitosis (Bostick et al., 2007; Sharif et al., 2007), are downregulated in early PGCs (Kurimoto et al., 2008), and components of the active demethylation pathways such as the hydroxylase *Tet1* and members of the base excision repair pathway are expressed (Hajkova et al., 2010). This is consistent with genetic studies which show that deficiency of the deaminase *Aid* (Popp et al., 2010) or the glycosylase *Tdg* (Cortellino et al., 2011) results in defects in methylation erasure in PGCs. Hence, the current thinking is that a combination of passive and active demethylation pathways is probably operating in PGCs, possibly in a context-dependent manner (Feng et al., 2010; Saitou et al., 2011; Hackett et al., 2012b).

The biological purposes and outcomes of epigenetic reprogramming in the germline are also not fully understood. Clearly, parental imprints need to be reprogrammed for normal development to occur in the next generation. Is reprogramming in PGCs really linked to pluripotency, and if so, why? Is most epigenetic information erased in germ cells so as to prevent the inheritance of acquired epigenetic information across generations? And are transposons resistant to reprogramming, or conversely, widely expressed in germ cells because of reprogramming?

We recently initiated studies for the genome-wide mapping of DNA methylation in PGCs using unbiased BS-Seq (Popp et al., 2010). Further optimization of the technique allowed us to include earlier stages of PGCs, and here we describe a systematic study of BS-Seq and RNA-Seq of key stages of PCG development, which provides a framework for the understanding of epigenetic reprogramming, pluripotency, and transgenerational epigenetic inheritance.

## Results

**Figure 1:**
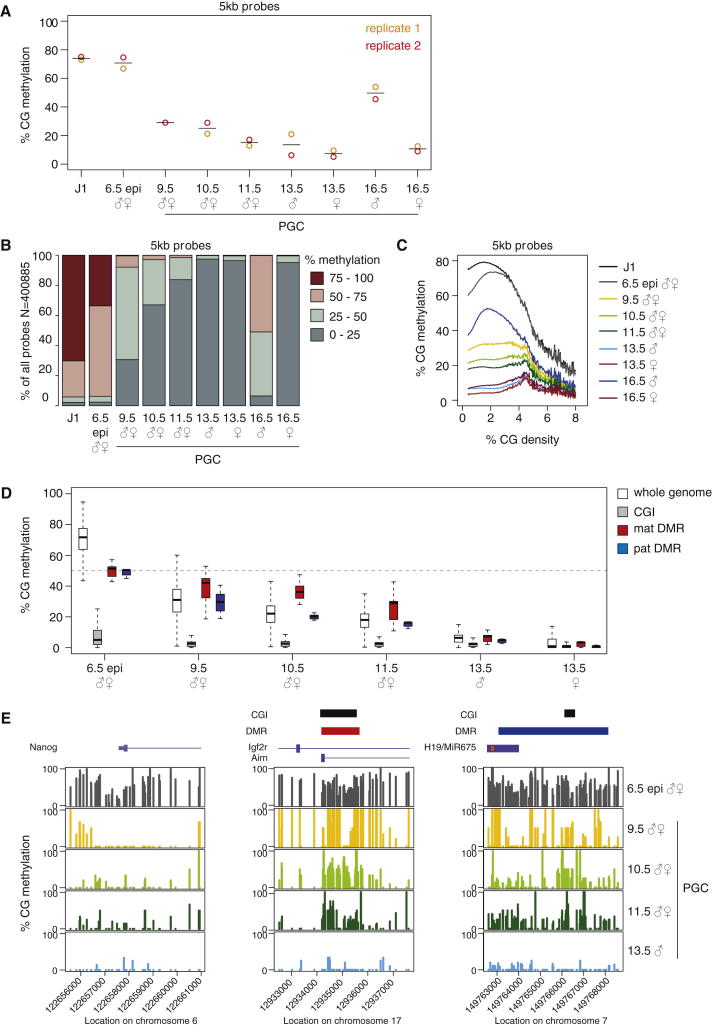
Demethylation Dynamics in PGCs (A) Global CG methylation levels for each data set assessed by 5 kb tiling probes. Open circles represent each data point; lines represent the median value for the two samples per time point. Note that the two replicates of the J1 data point are technical replicates, and all others are biological replicates from pooled samples. Note that E9.5 PGCs are already fairly hypomethylated followed by a further gradual loss of methylation toward E13.5. De novo methylation is only observed at E16.5 in male PGCs. See also Figure S1 and Table S1. (B) Distribution of CG methylation levels across the genome (5 kb probes). Note that the loss of methylation in PGCs is observed across the entire percentile distribution. (C) Correlation between CG density and methylation levels. (D) Methylation levels for the whole genome (5 kb probes), CGIs, and maternal and paternal DMRs (DMR coordinates were taken from E12.5 embryos [Tomizawa et al., 2011]). The dashed line indicates the expected 50% methylation levels for a germline DMR. Note that maternal DMRs retain more methylation than the rest of the genome during global loss of methylation. Outliers are not shown. (E) Example plots for *Nanog* promoter (left), maternally methylated *Igf2r* DMR (middle), and paternally methylated *H19* DMR (right). Each bar represents a single CG dinucleotide. Note that while the Nanog promoter shows early demethylation kinetics, CGIs within imprint DMRs undergo delayed demethylation. See also Figure S1.

PGCs are induced by external signals in the epiblast around E6.5 and first arise as a small group of about 40 cells in the proximal epiblast at E7.25 (Saitou, 2009). We therefore decided to profile E6.5 epiblast cells, as these are expected to have a primed epigenetic state characteristic of nascent PGCs. At E9.5, a small population of about 200 PGCs starts to migrate through the hindgut endoderm and reaches the gonadal anlagen at E10.5–E11.5 (Saitou, 2009). Using an *Oct4-Gfp* transgene (on a C57Bl/6J background) (Yoshimizu et al., 1999), we isolated PGCs at E9.5, E10.5, E11.5, E13.5, and E16.5. For each time point, PGCs from 10–30 embryos were pooled, and at E13.5 and E16.5, male and female PGCs were profiled separately. BS-Seq libraries were prepared from two independent samples of each time point, and two independent sequencing runs for a J1 embryonic stem cell (ESC) (129S4/SvJae) BS-Seq library were performed as well (Figure 1A). To assess bisulfite conversion efficiency, we measured CHH (H = C/A/T) methylation levels for 1 kb tiling probes across the genome and found that more than 60% of all 1 kb probes for each sample revealed 100% conversion, indicating high conversion efficiency (see Figure S1A online). RNA-Seq libraries were prepared from one pooled sample per time point. Table S1 summarizes the outcomes of the Illumina sequencing runs of all BS-Seq and RNA-Seq libraries.

### Methylation Erasure Occurs in Two Distinct Phases

In the E6.5 epiblast, the overall methylation level at CG dinucleotides was 71% similar to the values observed for J1 ESCs (74%) and to values reported for somatic and ESCs and the E6.5 postimplantation embryo (Hajkova et al., 2002; Lister et al., 2009; Laurent et al., 2010; Stadler et al., 2011; Smith et al., 2012) (Figure 1A). In E9.5 PGCs, methylation levels were already reduced to 30%, which means that the bulk of methylation erasure in PGCs occurs prior to E9.5. This is in line with previous reports using IF and locus-specific bisulfite sequencing (Seki et al., 2005; Guibert et al., 2012) but differs from the expectation that global erasure of methylation marks occurs concomitantly with imprint erasure from E11.5 to E13.5 (Reik et al., 2001). From E9.5, methylation levels were reduced gradually to about 15% in E11.5 PGCs, with a further drop to 14% and 7% in male and female E13.5 PGCs, respectively. The global loss of methylation affects all methylation levels (Figure 1B) and is mirrored by the loss of the correlation between CG density and methylation levels across all time points (Figure 1C). It is noteworthy that no de novo methylation was observed between E6.5 and E13.5 in any of the PGC samples, indicating that global demethylation is a unidirectional process (Figure S1B). In female PGCs the low levels of methylation at E13.5 persist to E16.5 with cells being in meiotic arrest (Saitou, 2009), while male E16.5 PGCs show evidence of robust de novo methylation with an increase to about 50% methylation (Figures 1A and 1B, and Figure S1B).

**Figure 2:**
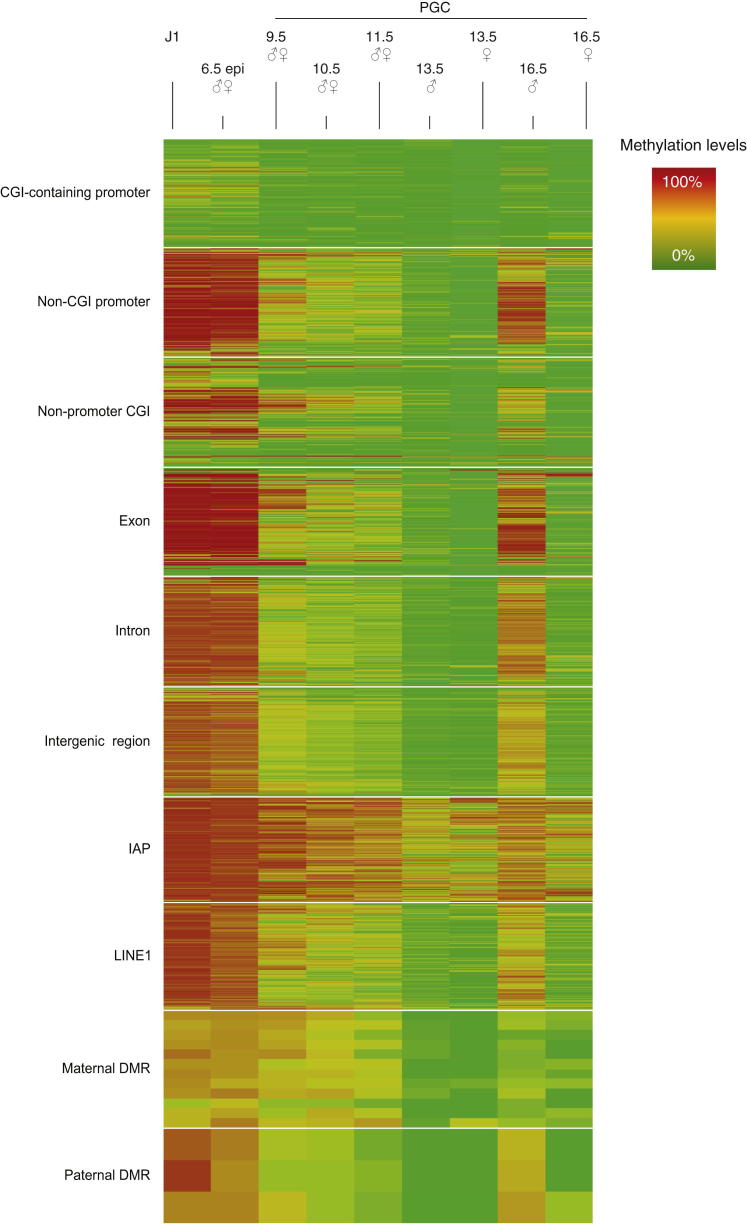
Methylation Heatmap for Various Genomic Features Each line represents a single probe within the indicated feature. High methylation levels are shown in red, low methylation levels are shown in green. Of all features analyzed, IAPs seem to retain most methylation at all time points, while all other features undergo substantial reprogramming.

The early phase of methylation erasure prior to E9.5 is truly global, affecting promoters, CpG islands (CGIs) (Deaton and Bird, 2011; Jones, 2012), introns, exons, and intergenic sequences (Figure 2). Promoters of genes that are expressed early in PGC development, such as *Nanog*, are demethylated during this phase (Figure 1E). However, there are a number of distinct sequence classes in which methylation marks are largely maintained during this early phase of methylation loss, and demethylation of these regions is only completed once PGCs enter the genital ridges from E10.5 (late demethylaters). These include DMRs of imprinted genes and particularly the maternal ones (Figures 1D, 1E, and 2, and Figure S1C). Closer inspection shows that sequences surrounding the CGIs in DMRs are also demethylated relatively early and that it is specifically the CGIs that are resistant to demethylation until the late stages of PGC development (Figure 1E and Figure S1C). This effect is less pronounced for paternal DMRs, which may be connected to their lower CG content (Schulz et al., 2010).

**Figure 3:**
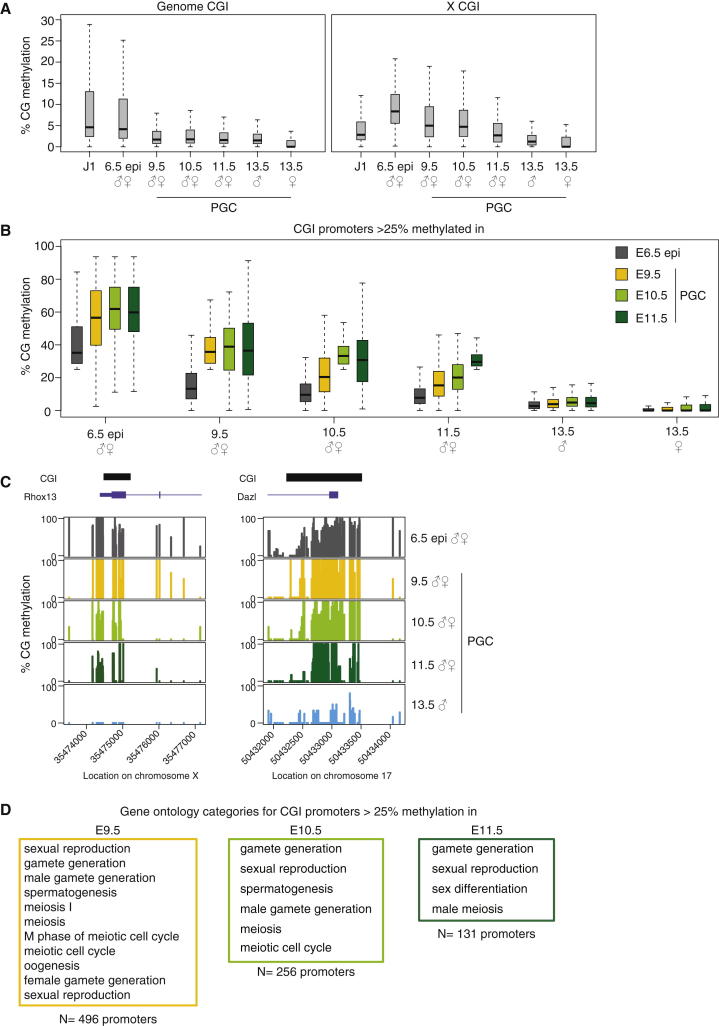
CGIs with Late Demethylating Kinetics (A) Demethylation kinetics of CGIs across the genome (left) or on the X chromosome (right). Note that X-linked CGIs undergo slower demethylation than CGIs for the rest of the genome. Outliers are not shown. See also Figure S2. (B) Demethylation kinetics for CGI-containing promoters selected with >25% methylation in E6.5 epiblast and E9.5, E10.5, and E11.5 PGCs. These promoters show consistently higher methylation levels across all time points analyzed. See also Table S2, Table S3, Table S4, and Table S5. (C) Example plots for an X-linked CGI (left) and a CGI-containing promoter with slow demethylation kinetics. Methylation marks seem to be retained especially around the CGI. Each bar represents a single CG dinucleotide. See also Figure S2. (D) Gene ontology categories with a Bonferroni-corrected p value < 0.05 for promoters selected for >25% methylation in E9.5, E10.5, and E11.5 PGCs. CGI promoters of genes selected for higher methylation levels between E9.5 and E11.5 seem to have a functional connection. The number of genes in each group is indicated.

Another class of late demethylater CGIs is found on the X chromosome (Figure 3A) (Brockdorff, 2011). The X-linked delayed demethylation is specific to CGIs, as the demethylation dynamics for the X chromosome as a whole mirror those of the genome globally (Figure S2A). It is noteworthy that CGIs on the X chromosome show elevated methylation levels in the E6.5 epiblast, as this is a pooled sample from male and female cells and thus includes a reduced but undefined number of inactivated X chromosomes contributed by female cells (Figure 3A). In an exclusively female epiblast, we expect 50% methylation for X linked CGIs, and this was observed for two female epiblast stem cell lines, which are derived from female epiblast (T. Hore, personal communication). The delayed demethylation kinetics for X-linked CGIs is significant because while it is known that early PGCs inherit a randomly inactivated X chromosome from the epiblast (Sugimoto and Abe, 2007), whether this involves methylation of CGIs with subsequent demethylation was unknown. Our data suggest that methylation at CGIs on the X chromosome is actively maintained during global methylation loss and results in a slow and gradual demethylation pattern, which is consistent with the gradual reactivation of X-linked genes over a prolonged period from E7.5 to E14.5 (Sugimoto and Abe, 2007).

We next identified a group of promoter CGIs that were demethylated with the same delayed kinetics as DMRs and retained more than 25% methylation for each time point prior to E13.5 (Figure 3B and Table S2, Table S3, Table S4, and Table S5). This cutoff was selected as it includes all of the DMRs, which are largely resistant to demethylation until E11.5, but excludes the vast majority of genomic CGIs, which exhibit less than 25% methylation at these time points (Figure 1D and Figure S2B). Notably, this group is associated with genes specifically involved in meiosis and gamete generation (Figure 3D), which in general are only transcribed in germ cells and methylated in most if not all somatic tissues (data not shown; Maatouk et al., 2006; Borgel et al., 2010; Hackett et al., 2012a). As observed for DMRs and X-linked CGIs, CGs in the neighborhood of these CGI promoters became demethylated in early PGCs, while the CGIs themselves retained methylation until E11.5 and became demethylated thereafter (Figure 3C, Figure S2C).

Thus, it seems that a select group of CGIs actively maintain methylation marks during the global loss of methylation that occurs in early PGCs. This is reminiscent of how methylation at some DMRs is maintained during global methylation loss in the early embryo by the zinc finger protein Zfp57 (Li et al., 2008). We found that late-demethylating CGI promoters were also substantially enriched for Zfp57 binding sites in ESCs (Quenneville et al., 2011) (Figure S2D); this preliminary finding suggests that Zfp57 might play a role in maintaining methylation marks at some CGIs during global loss of methylation in PGC development as it does for some DMRs during global methylation erasure in the early embryo.

### Mechanisms for Transgenerational Epigenetic Inheritance

**Figure 4:**
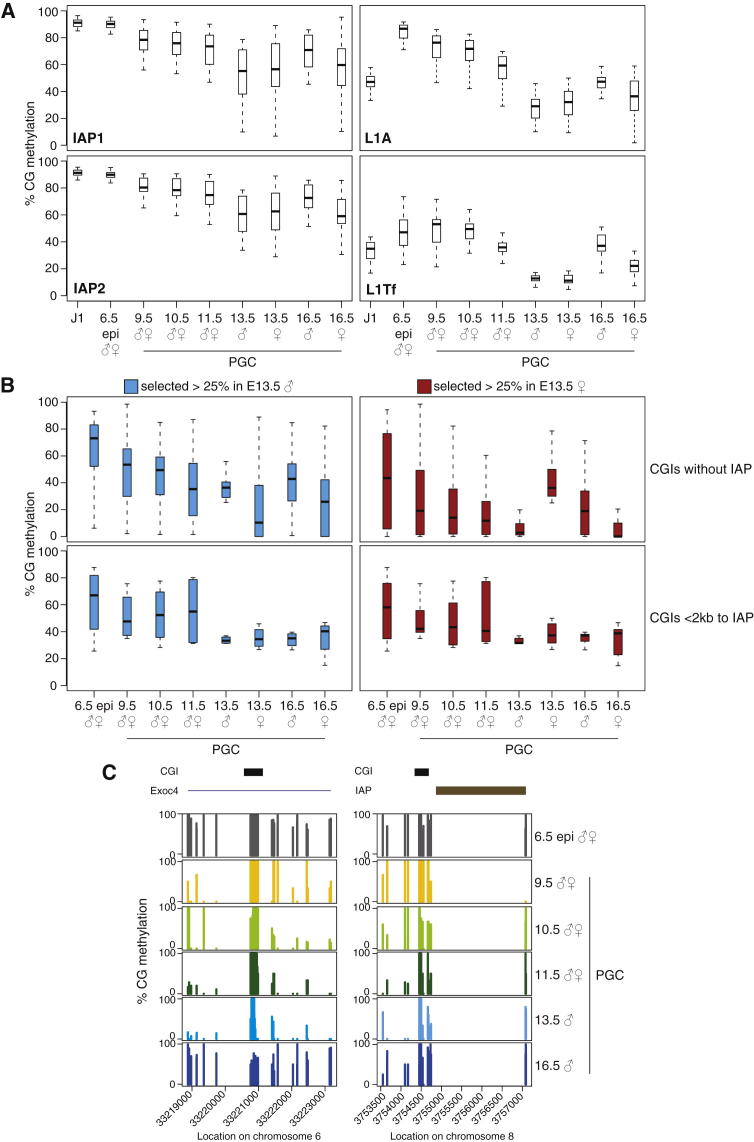
Demethylation Resistance (A) Methylation levels for CG dinucleotides within the consensus sequence of IAP1 (top left), IAP2 (bottom left), LINE1A (top right), and LINE1Tf (bottom right). Note that these elements retain substantial levels of methylation across all time points. Outliers are not shown. See also Figure S3. (B) Methylation levels for resistant CGIs selected with >25% methylation in E13.5 male (left) and female PGCs (right) without an IAP in close proximity (top) or near an IAP (bottom). Note that resistant CGIs with a distance of <2 kb to an IAP show consistently higher methylation levels across all time points, while CGIs without the presence of an IAP show variable methylation levels across all time points. See also Figures S3–S5. (C) Example figures for resistant CGIs without an IAP in close proximity (left) or with an IAP nearby (right). Each bar represents a single CG dinucleotide.

PGCs are in an extremely hypomethylated state at E13.5; however, a small amount of methylation is retained. We examined how the remaining methylation at E13.5 is distributed across the genome. We confirmed that as a sequence class only intracisternal A particles (IAPs) remained substantially methylated across all stages analyzed, while other elements such as LINE1s as well as SINEs retain small amounts of methylation at E13.5 but are largely reprogrammed (Figure 4A, Figure S3A). The resistance of IAPs against demethylation is particularly true for the consensus sequence of the monomer repeat within the long terminal repeat (LTR) of IAP1 and IAP2, two distinct classes of these aggressively transposing elements, while the 5′UTR of LINE1Tf and LINE1A elements, which have been extensively studied in ESCs (Ficz et al., 2011), undergoes significant demethylation (Figure 4A).

We investigated if any single-copy regions in the genome were resistant to demethylation. We identified resistant CGIs and non-CGI promoters that remained methylated (with a cutoff of 25% methylation) in male or female E13.5 PGCs (numbers are shown in Figure S3B). CGIs located close to an IAP showed consistently high methylation levels throughout all developmental stages, while CGIs without an IAP showed more variable resistance to erasure (Figure 4B, see below). It is noteworthy that resistant CGIs with an IAP were rare (Figure S3B) and that IAPs were more frequently found at resistant non-CGI promoters (Figures S3C and S4A). In fact, these non-CGI promoters are resistant to demethylation as a function of their distance from the IAP (Figure S4B). This is in line with previous reports (Guibert et al., 2012) and suggests that the genomic context or chromatin environment of IAPs can confer resistance to erasure on neighboring elements. Alternatively, prevention of demethylation of IAPs including their surrounding sequences may be a protective mechanism of the genome to avoid activation of these potentially mutagenic elements in the germline.

CGIs (and non-CGI promoters) that were not located close to an IAP were more variable in their resistance to demethylation (Figure 4B and Figure S5). However, some of these variably erased CGIs (VECs) remained methylated at all stages, including in mature oocytes and sperm (Figure S5A), for example a CGI in the *Exoc4* gene which is associated with type 2 diabetes and involved in insulin-stimulated glucose transport (Inoue et al., 2003) (Figure 4C). We have extended this analysis to a number of publicly available data sets from sperm, oocyte, two-cell embryo, ICM, and ESCs (Stadler et al., 2011; Kobayashi et al., 2012; Smith et al., 2012) (Figure S5B). Importantly, we observe that a substantial proportion of VECs retain significant methylation levels in various data sets, suggesting that VECs might be carriers of epigenetic inheritance transgenerationally. Interestingly, more of these CGIs were found methylated in sperm than in oocyte (Figure S5), implying that there may be a bias for such VECs to escape reprogramming in the male germline. These CGIs may be candidates for short-term transgenerational inheritance in mammals, which seems variable in its persistence and hence heritability (Jimenez-Chillaron et al., 2009; Carone et al., 2010; Ng et al., 2010; Daxinger and Whitelaw, 2012).

### Complex Mechanisms of Demethylation

The dynamics of global methylation erasure observed in our BS-Seq data sets shows that demethylation takes place over a prolonged period from before E9.5 to E13.5, during which PGCs undergo several cell divisions and hence cycles of DNA replication (Seki et al., 2007). Thus, we investigated if DNA demethylation in PGCs could be the result of a passive loss of methylation due to a lack of methylation maintenance at DNA replication. In such a scenario, methylation marks on the parental strand do not get copied onto the newly synthesized strand resulting in a hemimethylated product, which then becomes further diluted by continued replication and eventually results in complete hypomethylation.

**Figure 5:**
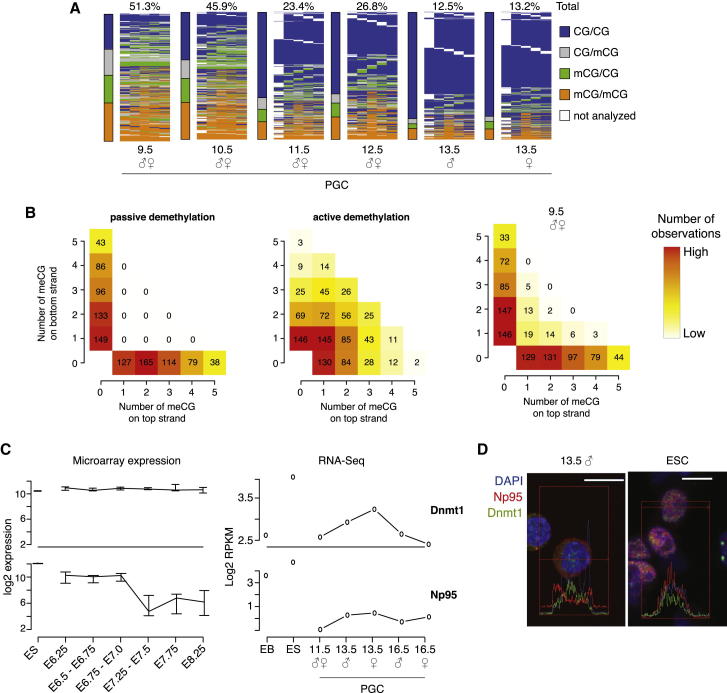
Mechanisms for Demethylation (A) Hairpin bisulfite heatmap of LINE1Tf. Total methylation levels are shown at the top. For each time point analyzed, each column represents one CG dyad along the LINE1Tf consensus sequence, and each row represents one sequencing read. The bars next to the heatmap represent the average distribution of fully methylated, hemimethylated, and unmethylated sites. Note that E9.5 and E10.5 PGCs have high levels of hemimethylated sites, which are then reduced to almost-complete hypomethylation at E13.5. (B) Shown is the distribution of methylated CG dinucleotides (meCG) at hemimethylated sites across the top (x axis) and bottom (y axis) strands of the LINE1Tf consensus sequence assessed by hairpin bisulfite sequencing. The LINE1Tf consensus sequence contains five CG dinucleotides, and the numbers 0–5 on the axis refer to the amount of meCGs on each strand and contain no position information. The values in the heat diagram represent the number of instances with the respective number of meCGs observed on the top and bottom strands. Shown is a simulation of the distribution of meCG within hemimethylated sites in the case of passive DNA demethylation (left) and active demethylation (middle). Note that with passive demethylation, all meCGs are located on the top strand, while the bottom strand is completely unmethylated and contains 0 meCGs and vice versa. For active DNA demethylation, a strand-independent distribution was simulated that leads to methylated and unmethylated CGs randomly distributed across both strands. The hairpin bisulfite data for E9.5 PGCs are shown in the right panel, and there is a strong strand bias for meCGs toward either top or bottom strand highly similar to the outcome for the simulation of passive DNA demethylation. Instances with meCGs distributed across both strands are rare in E9.5 PGCs. See also Figure S6. (C) Expression analysis of the DNA methylation machinery. Single-cell microarray data for ESCs (Vincent et al., 2011) and PGCs (Kurimoto et al., 2008) were reanalyzed (left, see the Experimental Procedures for more detail). RNA-Seq data for ESC and embryoid body (EB) (Cloonan et al., 2008) and PGCs of various time points are shown on the right. Whiskers represent the interquartile range of variation between replicates. Note that while *Dnmt1* is continuously expressed, *Np95* is transcriptionally downregulated in early PGCs. See also Figure S7. (D) Immunofluorescence staining for DNA (blue), Dnmt1 (green), and Np95 (red). Shown are immunostainings and RGB profiles created with Zeiss LSM software. Scale bars represent 10 μM in all images, and where RGB profiles are shown, the red line across a cell represents the midline along which the signal intensity is traced for each pixel and the profile is plotted below. Shown are stainings for Np95 and Dnmt1 in E13.5 male PGCs and E14 ESCs. In cycling PGCs, Dnmt1 localizes to the nucleus while Np95 is preferentially located in the cytoplasm. In ESCs, both Dnmt1 and Np95 localize to the nucleus. This suggests that in ESCs, the subcellular localization of Dnmt1 and Np95 is linked during S phase, while this dynamic pattern may be uncoupled in PGCs. See also Figure S7.

To gain more detailed molecular insights into the dynamics of demethylation, we carried out hairpin bisulfite high-throughput sequencing of the LINE1Tf 5′UTR. Hairpin bisulfite sequencing keeps the two original DNA strands together, allowing an assessment of full versus hemimethylation and demethylation at each CG (Arand et al., 2012). There was a substantial amount of hemimethylated CG sites in PGCs at E9.5 and E10.5, which was then reduced to the fully unmethylated state by E13.5 (Figure 5A). It is noteworthy that within sequences that were found to be hemimethylated, methylated CG dinucleotides were almost exclusively located on the same strand, and instances of hemimethylated sequences with methylated CG dinucleotides on both strands were rare (Figure 5B and Figure S6). Over the time course analyzed, the number of methylated CGs is drastically reduced toward E13.5, but the strand bias is preserved in all data sets (Figure S6). These dynamics are consistent with a predominantly passive demethylation mechanism with a minor contribution by active mechanisms.

The entire de novo methylation system including *Dnmt3a*, *Dnmt3b*, and *Dnmt3L* is transcriptionally silenced during this period (Hajkova et al., 2002; Kurimoto et al., 2008), consistent with our observations revealing a complete lack of de novo methylation until E16.5 in male PGCs, at which stage *Dnmt3a* and *Dnmt3L* show a burst of transcription (Figure S6A). Furthermore, while *Dnmt1* is expressed (Figure 5C) and localized in the nucleus (Hajkova et al., 2002) (Figure 5D), *Np95* is transcriptionally downregulated (Kurimoto et al., 2008; Figure 5C), and importantly we find that the remaining protein seems to be largely excluded from the nucleus in replicating PGCs, while Dnmt1 is not as confirmed by EdU staining (Figure 5D and Figure S7B). In ESCs, Np95 and Dnmt1 are both located in the nucleus, while control stainings in Np95 KO cells show no background staining for Np95 (Figure S7C). The predominant cytoplasmic localization of Np95 in PGCs was observed for all time points analyzed (Figure S7D) and is independent of the cell-cycle stage of these cells. This apparent retention of Np95, but not of Dnmt1, in the cytoplasm of PGCs suggests that the canonical somatic pathway for methylation maintenance, which involves Dnmt1 targeting to the replication fork by Np95, may be disabled. Together with the lack of de novo methylation, this could contribute to the global loss of methylation in early PGCs. At the same time, the presence (and most likely noncanonical targeting) of Dnmt1 presumably allows maintenance of methylation at DMRs of imprinted genes and other sequences that undergo late demethylation, which is strongly reminiscent of the maintenance of methylation marks at DMRs in the early embryo during global loss of methylation (Saitou et al., 2011). *Tet1*, among other factors involved in active demethylation, is expressed during both early and late PGC development at low levels (Figure S7A) (Hajkova et al., 2010), and our analysis does not exclude the presence of additional active demethylation pathways.

### Reprogramming the Transcriptional Landscape of PGCs

The erasure of most 5mC from the genome raises questions of transcriptional regulation. Are there large-scale transcriptional activation and promiscuity? And importantly, is there a global link between epigenetic reprogramming and pluripotency?

**Figure 6:**
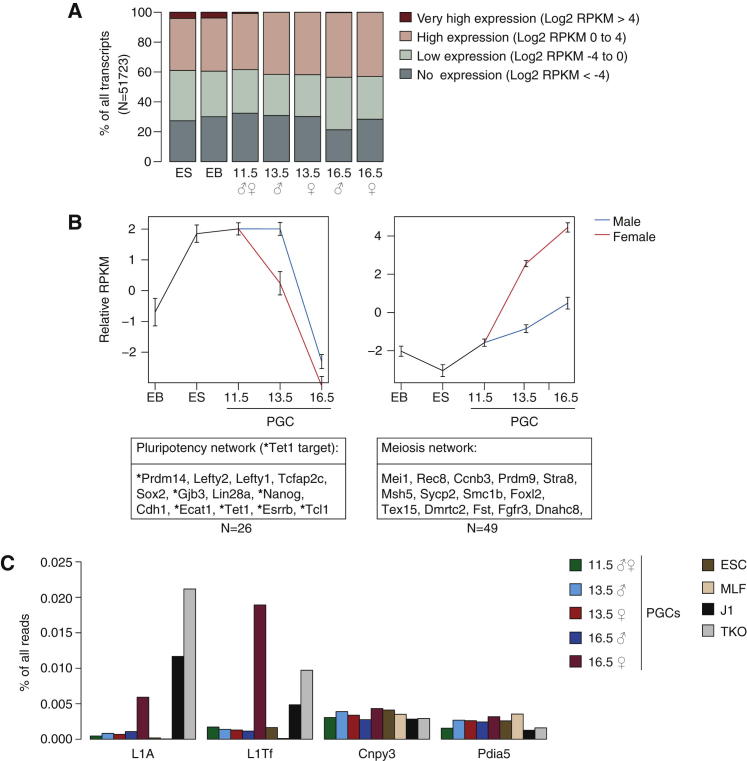
Dynamics of Transcriptomic Reprogramming (A) Distribution of expression values for the PGC RNA-Seq data sets. ESC and EB data sets were included for comparison (Cloonan et al., 2008). Even the most hypomethylated samples with E13.5 PGCs have an orderly expression program similar to that of all other PGC samples and the ESC and EB data sets. (B) Expression profiles for the pluripotency cluster (left) and the meiosis cluster (right). Error bars represent the standard deviation of measure across all probes within the cluster, and example genes are shown underneath for each cluster. Tet1 targets within the pluripotency cluster are highlighted by an asterisk. See also Figure S8 and Table S6. (C) Expression of LINE1s. Shown is the percentage of all RNA-Seq reads that map to the LINE1Tf and LINE1A consensus sequence and also as a comparison to the sequence of *Cnpy3* and *Pdia5*, two single-copy genes that are expressed at constant levels across the time course. Note that the two LINE1 elements have higher expression levels at E16.5 in female PGCs than in any other data set and also than the two single-copy genes. Results for RNA-Seq data from ESCs and mouse lung fibroblasts (MLFs) (Guttman et al., 2010), J1 (Ficz et al., 2011), and *Dnmt* TKO ESCs (KO for *Dnmt1*, *Dnmt3a*, and *Dnmt3b* [Karimi et al., 2011]) are shown for comparison.

We first looked at the complexity of the RNA-Seq transcriptome in PGCs in comparison to ESCs and somatic cells and found no fundamental shift in complexity, meaning that similar numbers of genes had high, intermediate, and low levels of transcription in all cell types including PGCs (Figure 6A). Hence the global loss of methylation at promoters (Figure 2) does not result in a profound shift in transcriptional regulation, indicating that a mechanism independent of DNA methylation promotes transcriptional control in reprogramming PGCs. Similarly, a loss of methylation over exons and introns (gene bodies) (Figure 2) was not accompanied by any shift in the transcriptional profile (Figure 6A). De novo methylation at E16.5 in male PGCs also seemed to be independent of transcriptional changes from E13.5 to E16.5 as the promoters of genes that increased or decreased in expression became de novo methylated at similarly high levels (Figure S8A). However, gene body methylation was positively correlated with transcription in E16.5 male PGCs (Figure S8B). This suggests that DNA methylation and transcription are largely uncoupled during methylation erasure in PGCs but show some degree of positive correlation when genome-wide methylation is restored, suggesting that the relationship between DNA methylation and transcription is complex (Jones, 2012).

Next we defined clusters of genes with a highly similar expression profile across the different stages of PGC development (see the Supplemental Experimental Procedures for details). We discovered 12 clusters of transcripts that changed in consistent ways over the time course analyzed (data not shown). The two largest clusters with 26 and 49 genes, respectively, revealed interesting sets of genes with functional importance for PGC development (Figure 6B and Table S6). The first cluster was highly enriched for transcription factors of the pluripotency network, which are fully expressed at E11.5 with a steep decline toward E16.5 (Figure 6B). The second cluster begins to be transcribed as the pluripotency network declines and corresponds to meiosis network genes (Figure 6B). Expression of these transcripts is particularly high in female PGCs from E13.5, which arrest in meiotic prophase at that time (Bowles and Koopman, 2010) (Figure 6B). Notably, the pluripotency cluster that we identified is particularly enriched for Tet1 targets (based on transcriptomics in *Tet1* knockdown or knockout ESCs and on Tet1 ChIP-seq data in ESCs) (Figure 6B) (Dawlaty et al., 2011; Ficz et al., 2011; Williams et al., 2011; Wu et al., 2011). Interestingly, the promoters of these transcripts associated with genes such as *Nanog* and *Prdm14* are methylated in E6.5 epiblast cells, and all become demethylated in PGCs by E9.5 (Figure 1E and Figure S8C), indicating that demethylation of these promoters may be connected with their activity in early PGCs. Transcription of the pluripotency network is then collectively silenced as female PGCs go into meiotic arrest and male PGCs into mitotic arrest around E13.5 (Bowles and Koopman, 2010). Expression of these genes is replaced by the network of meiosis- and germ-cell-function-related genes further driving PGCs toward germ cell fate (Figure 6B).

Lastly, we were interested to see if the substantial demethylation in LINE1 elements resulted in their transcriptional activation. Surprisingly, demethylation did not lead to general transcriptional activation of LINE1s in PGCs by E13.5 (Figure 6C). However, there was a specific transcriptional burst of LINE1 elements exclusively in female E16.5 PGCs, consistent with the possibility that LINE1 particles persist during oogenesis, leading to transposition events in early embryos (Figure 6C) (Kano et al., 2009). Nonetheless, it is unclear why this activation does not take place at E13.5, as methylation levels in female PGCs at E13.5 and E16.5 are similarly low. It seems that expression of repetitive elements does not consistently show an inverse correlation to DNA methylation, and additional mechanisms other than DNA methylation are in place to regulate LINE1 expression.

## Discussion

We have carried out a systematic study of genome-wide DNA methylation (BS-Seq) and transcription (RNA-Seq) across key stages of PGC development during which epigenetic reprogramming takes place. A similar study of methylation reprogramming in preimplantation embryos using RRBS-Seq has been recently published (Smith et al., 2012). Together these studies provide an advanced framework for the understanding of the dynamics of reprogramming in embryonic development and their biological outcomes. Our work provides four key insights. First, it defines two phases of demethylation in PGCs, global demethylation occurring early during their migration with the methylation of specific regions being actively maintained, and a second phase which occurs upon entry into the genital ridges and affects sequences carrying epigenetic memory. Second, global DNA demethylation in PGCs is consistent with contribution from a passive mechanism supplemented by active maintenance of methylation in specific regions, which ceases upon arrival in the gonads. Third, global erasure of methylation does not lead to promiscuous transcription including that of retrotransposons; instead the core pluripotency network is expressed at early stages of PGC development and is then replaced by expression of a meiosis and germ cell development network. Finally, we identify VECs that may act as carriers of short-term transgenerational epigenetic inheritance in mammals.

An important question that arises from the early demethylation dynamics of PGCs is whether these cells have somatic methylation levels to begin with. Earlier work using an antibody against 5mC to visualize DNA methylation suggested that E8.0 PGCs retain a signal intensity comparable to somatic cells, which diminishes subsequent to this stage (Seki et al., 2005). Bisulfite sequencing analysis of individual loci showed that E8.5 PGCs retain high levels of methylation at certain loci (Guibert et al., 2012). In addition, the presence of hemimethylated sites in LINE1 elements at E9.5 implies that these elements have undergone demethylation and thus are likely to have started out from epiblast-like methylation levels. This body of evidence strongly suggests that the earliest PGCs emerging in the E7.25 epiblast inherit a highly methylated genome characteristic of epiblast cells.

Indiscriminate genome-wide loss of methylation occurs early in PGC development and is accompanied by the transcriptional downregulation of the de novo methyltransferases (*Dnmt3a,b,L*) and also seems to involve the impairment of the methylation maintenance factor Np95. By contrast, DMRs in imprinted genes, CGI promoters of germ-cell-specific genes, and CGIs on the X chromosome have their methylation largely maintained during global methylation loss, and demethylation of these sequences is only completed once PGCs have entered the genital ridges. Interestingly, this suggests that the mechanisms of demethylation in PGCs and in preimplantation embryos share similarities including passive and active demethylation, with perhaps a key difference being the continuing protection from demethylation of imprinted DMRs by Zfp57 in preimplantation embryos and ESCs, which is lacking in PGCs (Li et al., 2008; Quenneville et al., 2011). Also, it is unclear at this point if methylation present in the PGC founder population is first converted into 5-hydroxymethylcytosine (5hmC) and then lost by subsequent passive demethylation, as BS-Seq data sets do not distinguish between 5mC and 5hmC, and current techniques that allow for this distinction require amounts of input material that are not currently applicable to PGCs (Booth et al., 2012; Yu et al., 2012). In addition, other demethylation mechanisms involving factors such as *Aid* and *Tdg* have been shown to play a role in DNA methylation reprogramming in PGCs (Popp et al., 2010; Cortellino et al., 2011), suggesting that active and passive mechanisms of demethylation work in concert to ensure robust epigenetic reprogramming in PGCs (Feng et al., 2010; Saitou et al., 2011; Hackett et al., 2012b).

Global demethylation in PGCs is not associated with promiscuous transcriptional activation. Indeed, LINE1 elements, which have been substantially demethylated by E13.5, are not transcribed at that stage, suggesting that other mechanisms for transcriptional repression of retrotransposons are in place, such as those provided by *Setdb1* and *Kap1* in ESCs (Rowe et al., 2010; Karimi et al., 2011). Early PGCs transcribe *Oct4*, *Nanog*, and slightly later, *Sox2*, consistent with the possibility that they activate at least part of the pluripotency transcription factor network (Surani et al., 2007). Indeed, our transcriptome analysis shows that from E11.5 to E13.5 the core pluripotency network is fully transcribed at similar levels as in ESCs, consistent with the capability of deriving EGCs from these stages of PGC development. Activation of the pluripotency network is associated with promoter demethylation (from E6.5 epiblast cells to E9.5 PGCs) and with demethylation of H3K27me3 by the histone demethylase Utx (Mansour et al., 2012). Without any change in genomic methylation patterns, this transcriptional program is extinguished by E16.5 and replaced by the meiosis network, especially in female PGCs (which are in meiotic prophase arrest). How the pluripotency network is silenced and the meiosis network activated in such a coordinated fashion remains to be elucidated.

Why is the full pluripotency network activated in PGCs when these cells subsequently undergo a defined differentiation program rather than the pluripotential one of the ICM cells? We suggest that while epigenetic reprogramming is tightly connected with the activation of the pluripotency network in PGCs and early embryos, similarly the expression of the pluripotency network may be linked to demethylation of some of the targets described here. Notably, the pluripotency network expressed in PGCs is enriched for Tet1 targets, and Tet1 itself could be responsible for demethylation of these factors. This is supported by the fact that these promoters are almost completely demethylated by E9.5, but further analysis of earlier stages is needed to confirm if these promoters become demethylated with even faster kinetics than the rest of the genome. These factors may have evolved to become demethylated by faster and more targeted mechanisms than passive loss of methylation. Hence pluripotency and reprogramming appear to be inextricably linked in PGCs as suggested for ESCs (Ficz et al., 2011).

While most DNA methylation is erased by E13.5, there are some notable exceptions. First, IAPs are the class of sequences most resistant to demethylation, as previously observed (Lane et al., 2003; Guibert et al., 2012), consistent with IAPs being the evolutionarily most recently acquired transposon family in the mouse genome, which is still potentially very active and hence needs to be suppressed by methylation in the germline. This property explains the transgenerational epigenetic inheritance of the viable yellow (*A*^*vy*^) and axin-fused (*Axin*^*Fu*^) mutant alleles in the mouse, which have arisen by insertion of an IAP LTR into the *agouti* or *fused* gene, respectively (Morgan et al., 1999; Rakyan et al., 2003). Indeed, CGIs in the neighborhood of an IAP (up to 2 kb away) are resistant to erasure. Importantly, we identified a number (89 and 176 in male and female PGCs, respectively) of CGIs outside of an IAP context in which DNA methylation was incompletely erased at E13.5 (Figure 4C shows the example of the *Exoc4* gene which is associated with type 2 diabetes and involved in insulin-stimulated glucose transport [Inoue et al., 2003]). Most of these CGIs are variably erased, meaning that their extent of erasure differs between stages, the sexes, and potentially between individuals. The molecular mechanism of transgenerational epigenetic inheritance is not known, but there are several examples of epigenetic heritability through the male germline (Daxinger and Whitelaw, 2012), which might be consistent with our observation that VECs are more resistant to erasure in male than in female PGCs. Characteristically, this type of epigenetic inheritance shows variable penetrance, and the phenotype is frequently lost after a short number of generations (Daxinger and Whitelaw, 2012). This makes VECs interesting candidates for transgenerational epigenetic inheritance of induced metabolic phenotypes, and perhaps more generally, for variations in phenotype that are not predicted by genotype (intangible variation).

## Experimental Procedures

### Sample Collection

All embryonic samples for library preparation were collected from timed matings of C57Bl/6J female mice. Embryos collected for the E6.5 epiblast samples were isolated and mechanically dissected, separating away all extraembryonic tissues, and pooled prior to DNA and RNA isolation. PGCs were isolated from timed mated females carrying the *Oct4-Gfp* transgene expressed in the developing gonad (Yoshimizu et al., 1999) on a C57Bl/6J background. PGCs from 10–30 embryos were pooled for each time point, and final PGC numbers ranged from 800 to 40,000. For E13.5 and E16.5 PGCs, male and female samples were collected separately, as gonads can be readily distinguished morphologically from E13.5. PGC samples were collected following collagenase digestion using a FACSAria cell sorter with >98% purity. J1 ESCs (129S4/SvJae) were grown on feeder cells under standard conditions as described previously (Ficz et al., 2011). Animal work carried out as part of this study is covered by a project license (to W.R.) under the 1986 animal (scientific procedures) act, and is further regulated by the Babraham Institute Animal Welfare, Experimentation, and Ethics Committee.

### BS-Seq Library Prep

The amount of input material for the BS-Seq libraries was between 5 ng and 50 ng genomic DNA. The input DNA was sonicated, and end repair and A-tailing were performed using the NEB Next kit according to the manufacturers’ instructions. Illumina’s Early Access Methylation Adaptor Oligo Kit was used for the adaptor ligation. The adaptor-ligated DNA was treated with sodium-bisulfite using the Imprint DNA Modification Kit from Sigma-Aldrich according to the manufacturer’s instructions for the two-step protocol. Bisulfite-treated DNA was amplified using PfuTurbo Cx Hotstart DNA Polymerase from Agilent Technologies with 14–18 cycles depending on the input amount. Size selection was performed by gel extraction for DNA fragments between 200 bp and 250 bp.

### RNA-Seq Library Prep

Between 30 ng and 100 ng total RNA was DNase treated with Ambion’s DNA-free Kit according to the manufacturer’s instructions. Enrichment for mRNA was performed using Dynabeads Oligo(dT)_25_ from Invitrogen in two subsequent steps of purification with fresh beads. The isolated mRNA was fragmented and converted into cDNA. For the library preparation, the NEB Next kit was used according to the manufacturers’ instructions. Illumina PE adapters were ligated onto the end-repaired and A-tailed cDNA. Libraries were amplified with 12 cycles and size selected by gel extraction for fragments between 200 bp and 250 bp.

### Immunofluorescence

Antibody staining against Np95 (Th10, gift from Haruhiko Koseki) and Dnmt1 (sc-20701, Santa Cruz Biotechnology) was performed as previously described (Santos et al., 2003) with modifications. PGCs were identified either by the presence of Oct4-Gfp or by staining for Stella. EdU incorporation was achieved by incubating gonads before staining (as per the manufacturer’s instructions–Invitrogen). Single optical sections were captured with a Zeiss LSM510 Meta microscope (63× oil immersion objective).

### Hairpin BS with PGC Collection

For the hairpin bisulfite analysis, PGCs were isolated from *Oct4-Gfp* transgenic embryos (Yoshimizu et al., 1999) at the desired time points (E9.5–E13.5). Isolated genital ridges were trypsinized, and single GFP-positive cells were collected manually using inverted fluorescence microscope Zeiss AxioVert 200M and micromanipulators TransferMan NK2 (Eppendorf). Each sample contained at least 40 PGCs. Hairpin bisulfite sequencing for LINE1Tf 5′UTR was carried out on a 454 sequencing platform as described previously (Arand et al., 2012).

### DNA Sequencing

Libraries were sequenced on either an Illumia GAIIx or an Illumina HiSeq using the default RTA analysis software. See Table S1 for the outcomes of the sequencing runs.

### Data Analysis

Computational methods are described in the Supplemental Experimental Procedures.

## Accession Numbers

Raw data from all libraries were deposited with the European Nucleotide Archive under the accession number ERP001953.

## Supplemental Information

Document S1. Figure S1, Figure S2, Figure S3, Figure S4, Figure S5, Figure S6, Figure S7, Figure S8, Supplemental Experimental Procedures, and Supplemental References

Table S1. Details for all Illumina Sequencing Runs, Relates to Figure 1 Final alignments are deduplicated for all BS-Seq samples. Unique alignments without deduplication are shown for the RNA-Seq samples.

Table S2. CGI Promoters with >25% Methylation in E6.5 Epiblast, Relates to Figure 3

Table S3. CGI Promoters with >25% Methylation in E9.5 PGC, Relates to Figure 3

Table S4. CGI Promoters with >25% Methylation in E10.5 PGC, Relates to Figure 3

Table S5. CGI Promoters with >25% Methylation in E11.5 PGC, Relates to Figure 3

Table S6. Transcripts within the Pluripotency and Meiosis Clusters, Relates to Figure 6
