# Supplementary material for: The Dynamics of Genome-wide DNA Methylation Reprogramming in Mouse Primordial Germ Cells
Source: Mol Cell. 2012 Dec 28;48(6):849–62. doi: 10.1016/j.molcel.2012.11.001 (PMC3533687; doi:10.1016/j.molcel.2012.11.001)
Supplement: Document S1. Figure S1, Figure S2, Figure S3, Figure S4, Figure S5, Figure S6, Figure S7, Figure S8, Supplemental Experimental Procedures, and Supplemental References [file mmc1.pdf]

Molecular Cell, Volume *48*

Supplemental Information

The Dynamics of Genome-wide DNA Methylation Reprogramming in Mouse Primordial Germ Cells

Stefanie Seisenberger, Simon Andrews, Felix Krueger, Julia Arand, Jörn Walter, Fátima Santos, Christian Popp, Bernard Thienpont, Wendy Dean, and Wolf Reik

Supplemental Information Inventory

Figure S1. Global methylation profiling, relates to Figure 1.

Figure S2. Promoters with slow demethylating kinetics, relates to Figure 3.

Figure S3. Resistance to demethylation, relates to Figure 4.

Figure S4. Local effect of IAPs on methylation resistance, relates to Figure 4.

Figure S5. Resistant CGIs in various datasets, relates to Figure 4.

Figure S6. Continued hairpin phasing analysis of the LINE1Tf consensus sequence, relates to Figure 5.

Figure S7. Continued analysis of passive demethylation pathways, relates to Figure 5.

Figure S8. Correlation analysis for transcription and DNA methylation, relates to Figure 6.

Table S1: Details for all Illumina sequencing runs, relates to Figure 1.

Table S2: CGI promoters with >25% methylation in E6.5 epiblast, relates to Figure 3.

Table S3: CGI promoters with >25% methylation in E9.5 PGC, relates to Figure 3.

Table S4: CGI promoters with >25% methylation in E10.5 PGC, relates to Figure 3.

Table S5: CGI promoters with >25% methylation in E11.5 PGC, relates to Figure 3.

Table S6: Transcripts within the pluripotency and meiosis clusters, relates to Figure 6.

Supplemental Information: Figure legends, table legends, extended experimental procedures, supplemental references, Figures S1 – S8.

Figure S1

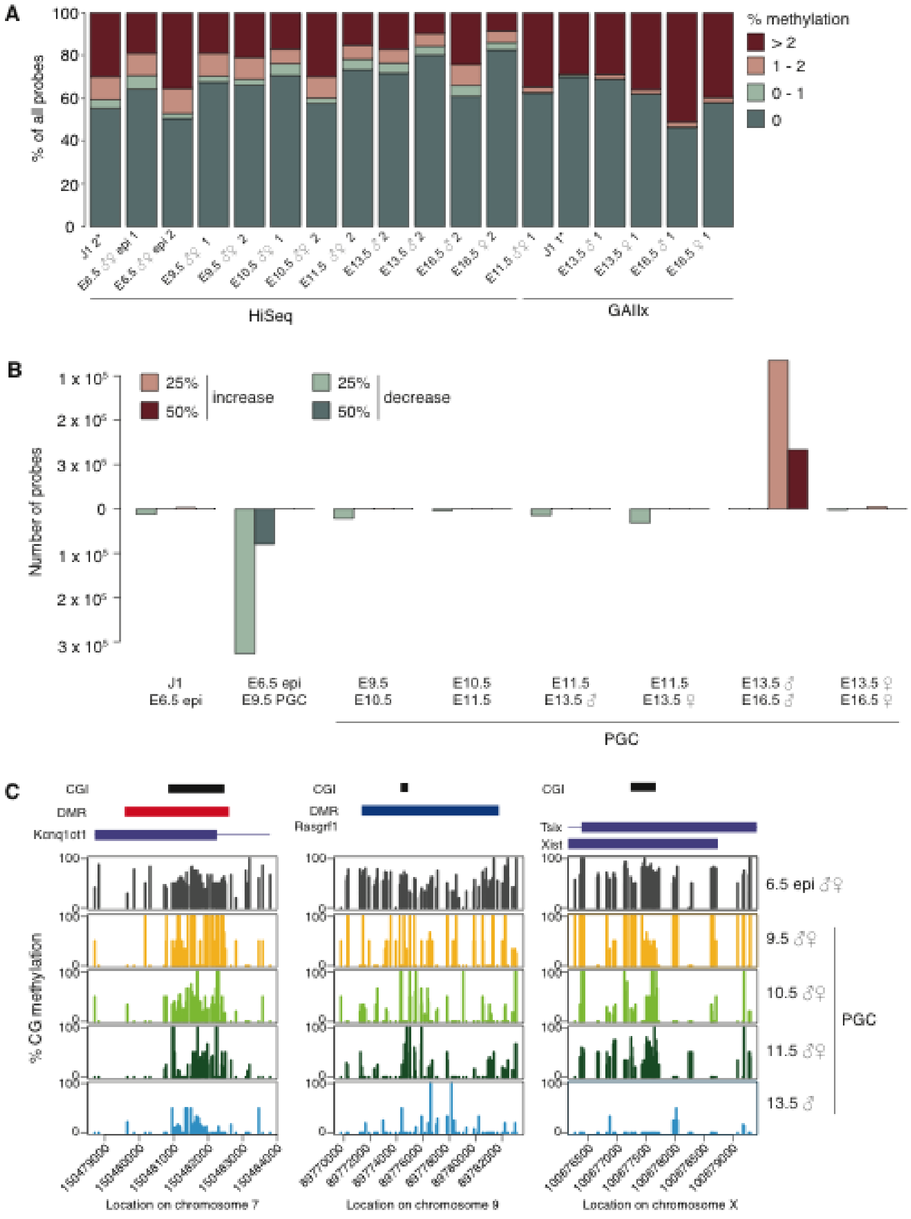
A
% of all probes
% methylation
> 2
1 - 2
0 - 1
0
HiSeq
GAIIx
B
Number of probes
25%
50%
increase
decrease
J1
E6.5 epi
E6.5 epi
E9.5 PGC
E9.5
E10.5
E11.5
E13.5 ♂
E13.5 ♀
E16.5 ♂
E16.5 ♀
PGC
C
CGI
DMR
Kcnq1ot1
Rasgrf1
Tsix
Xist
% CG methylation
6.5 epi ♂♀
9.5 ♂♀
10.5 ♂♀
11.5 ♂♀
13.5 ♂
PGC
Location on chromosome 7
Location on chromosome 9
Location on chromosome X

Figure S2

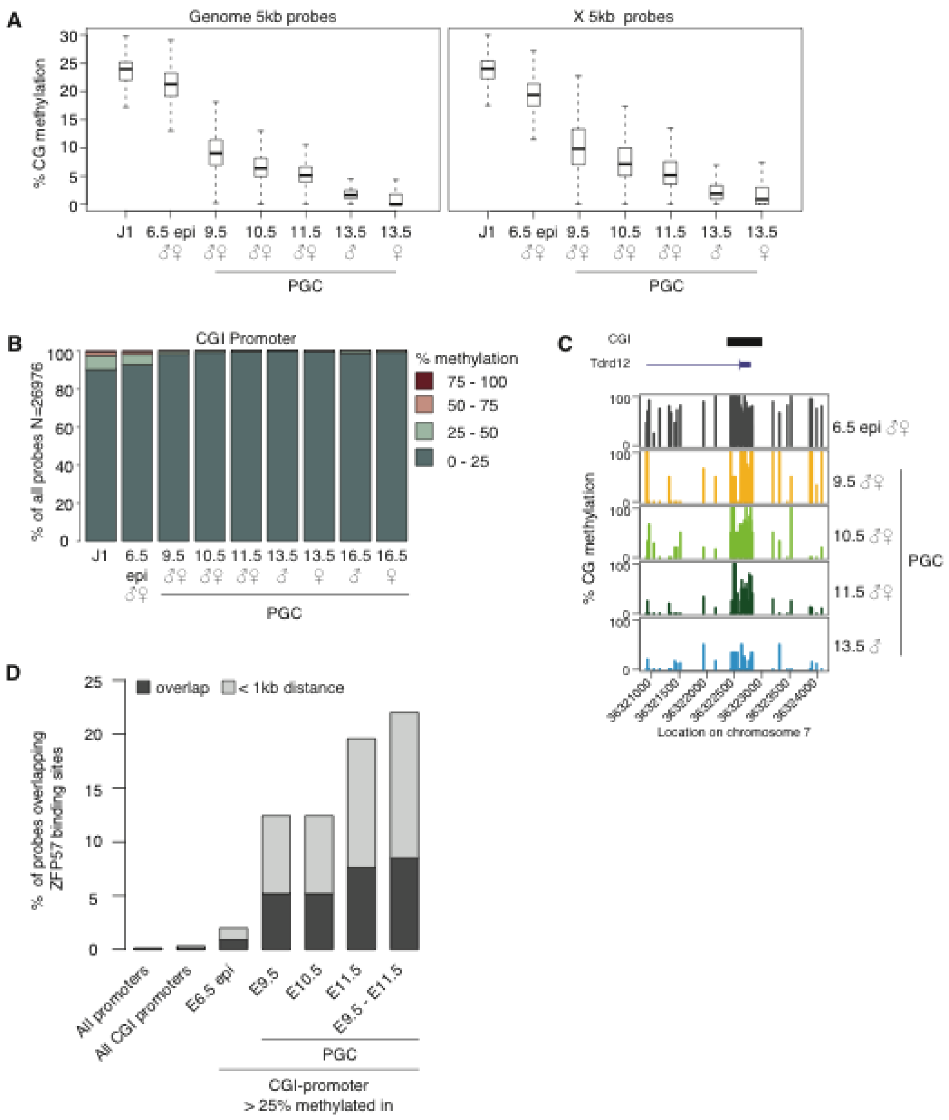
A
Genome 5kb probes
X 5kb probes
% CG methylation
J1
6.5 epi
9.5
10.5
11.5
13.5
PGC
B
CGI Promoter
% of all probes N=26976
% methylation
75 - 100
50 - 75
25 - 50
0 - 25
16.5
C
CGI
Tdrd12
% CG methylation
Location on chromosome 7
D
overlap
< 1kb distance
% of probes overlapping ZFP57 binding sites
All promoters
All CGI promoters
E6.5 epi
E9.5
E10.5
E11.5
E9.5 - E11.5
CGI-promoter
> 25% methylated in

Figure S3

A

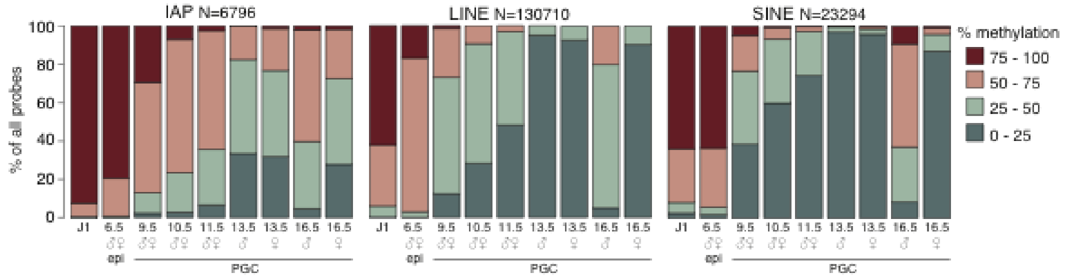

B

	13.5 ♂	13.5 ♀	Overlap ♂ & ♀
Non-CGI promoter without IAP	242	259	27
Non-CGI promoter < 2kb to IAP	44	26	20
CGI without IAP	89	176	22
CGI < 2kb to IAP	8	6	5

C

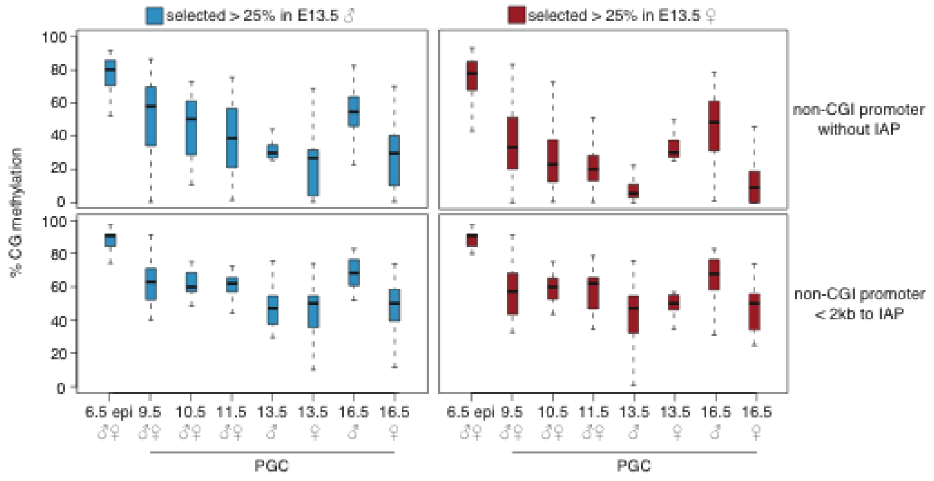

Figure S4

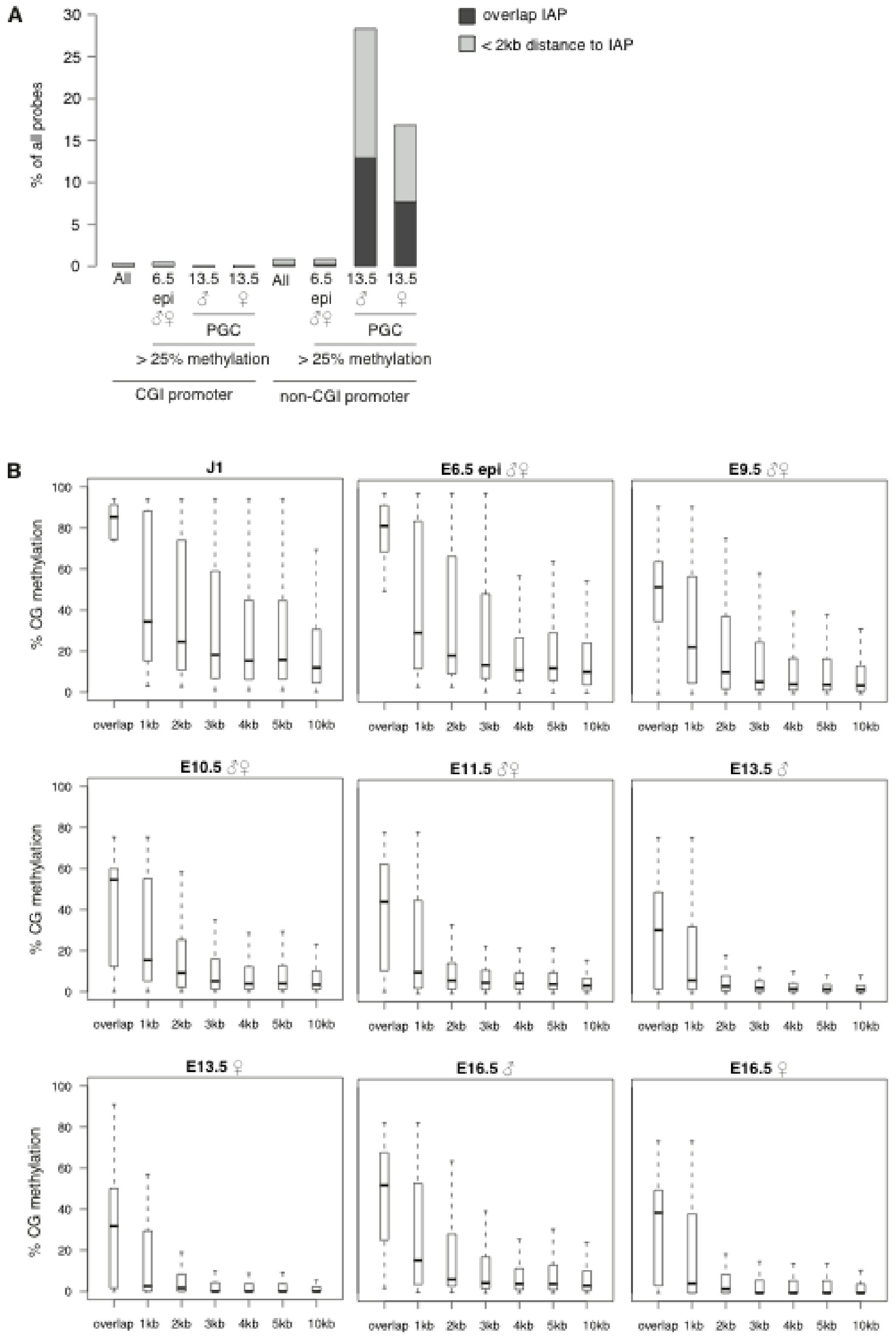
A
overlap IAP
< 2kb distance to IAP
% of all probes
All
6.5 epi ♂♀
13.5 ♂
13.5 ♀
PGC
> 25% methylation
CGI promoter
non-CGI promoter
B
J1
E6.5 epi ♂♀
E9.5 ♂♀
E10.5 ♂♀
E11.5 ♂♀
E13.5 ♂
E13.5 ♀
E16.5 ♂
E16.5 ♀
% CG methylation
overlap
1kb
2kb
3kb
4kb
5kb
10kb

Figure S5

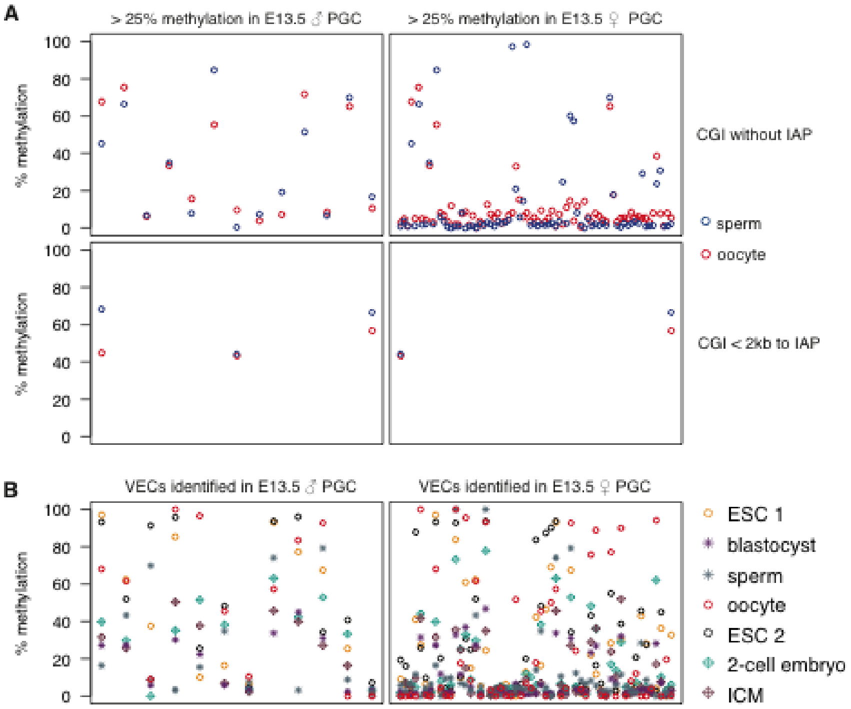

A
> 25% methylation in E13.5 ♂ PGC
> 25% methylation in E13.5 ♀ PGC
% methylation
CGI without IAP
sperm
oocyte
CGI < 2kb to IAP
B
VECs identified in E13.5 ♂ PGC
VECs identified in E13.5 ♀ PGC
ESC 1
blastocyst
sperm
oocyte
ESC 2
2-cell embryo
ICM

Figure S6

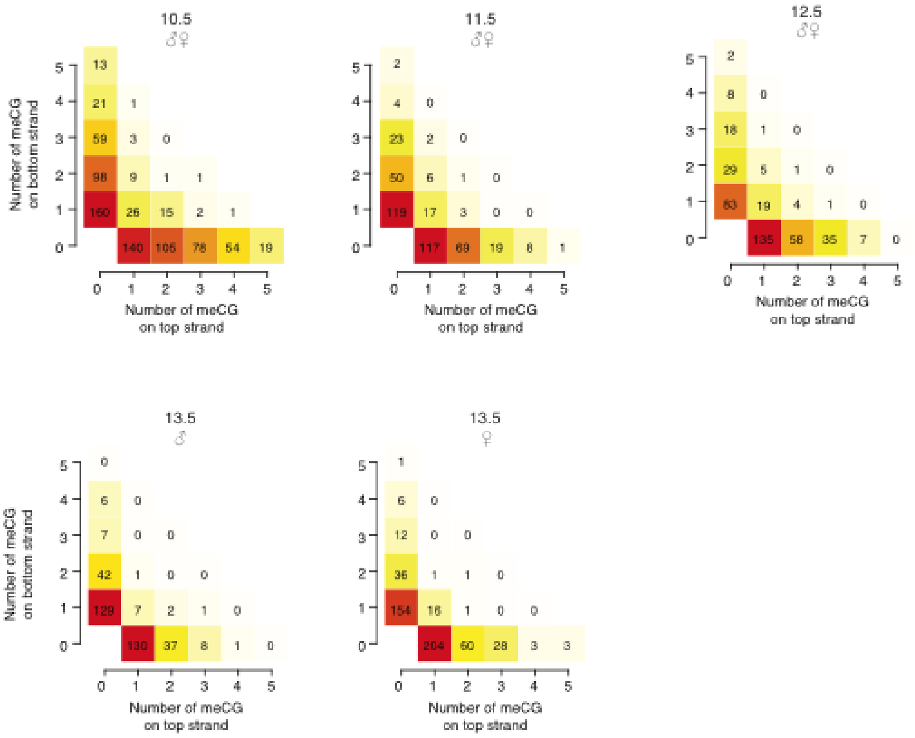
10.5
♂♀
11.5
♂♀
12.5
♂♀
13.5
♂
13.5
♀
Number of meCG
on bottom strand
Number of meCG
on top strand

Figure S7

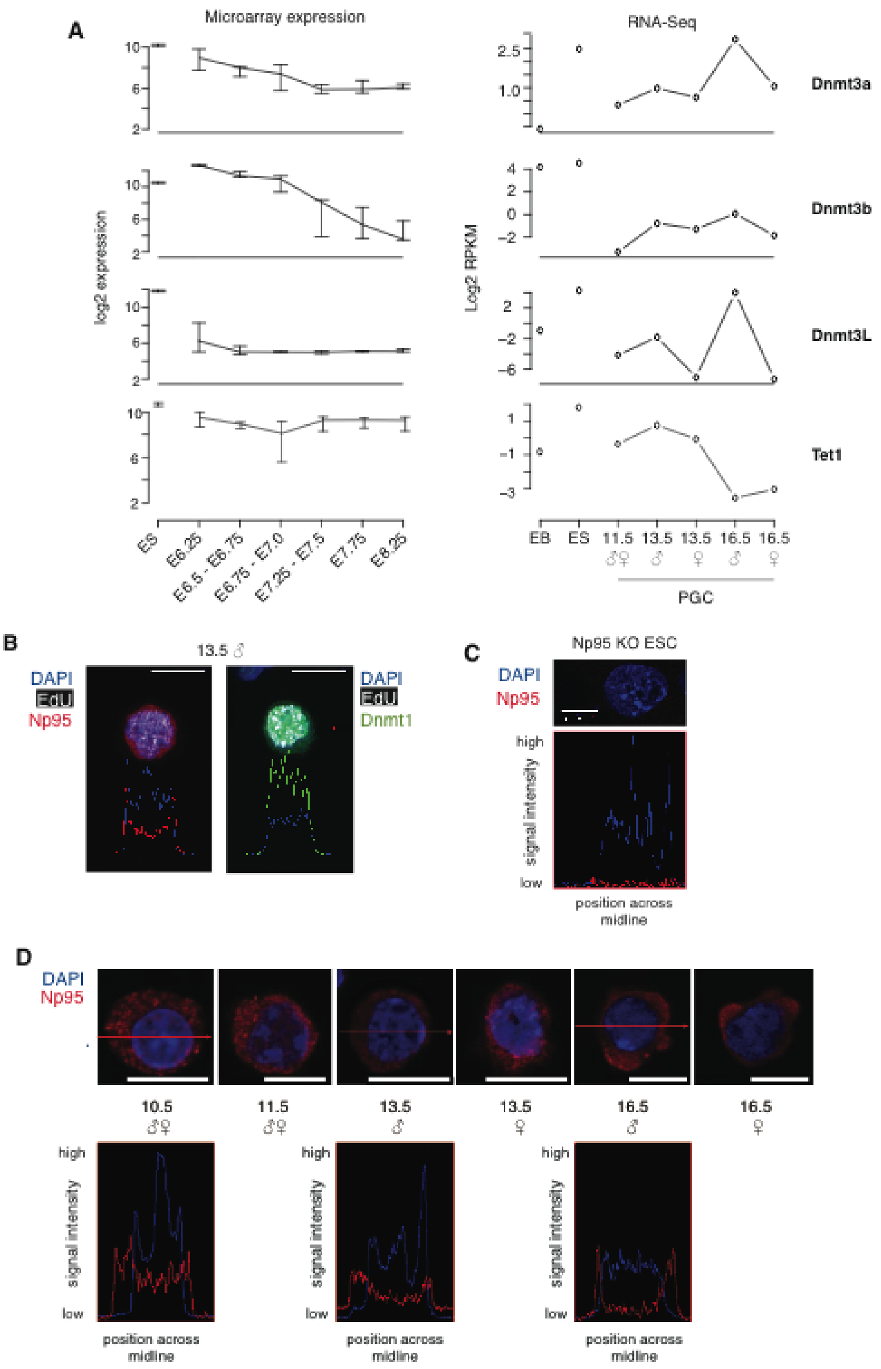

A
Microarray expression
RNA-Seq
log2 expression
Log2 RPKM
Dnmt3a
Dnmt3b
Dnmt3L
Tet1
ES
E6.25
E6.5 - E6.75
E6.75 - E7.0
E7.25 - E7.5
E7.75
E8.25
EB
ES
11.5
13.5
16.5
PGC
B
13.5 ♂
DAPI
EdU
Np95
Dnmt1
C
Np95 KO ESC
high
low
signal intensity
position across midline
D
10.5
11.5
13.5
16.5

Figure S8

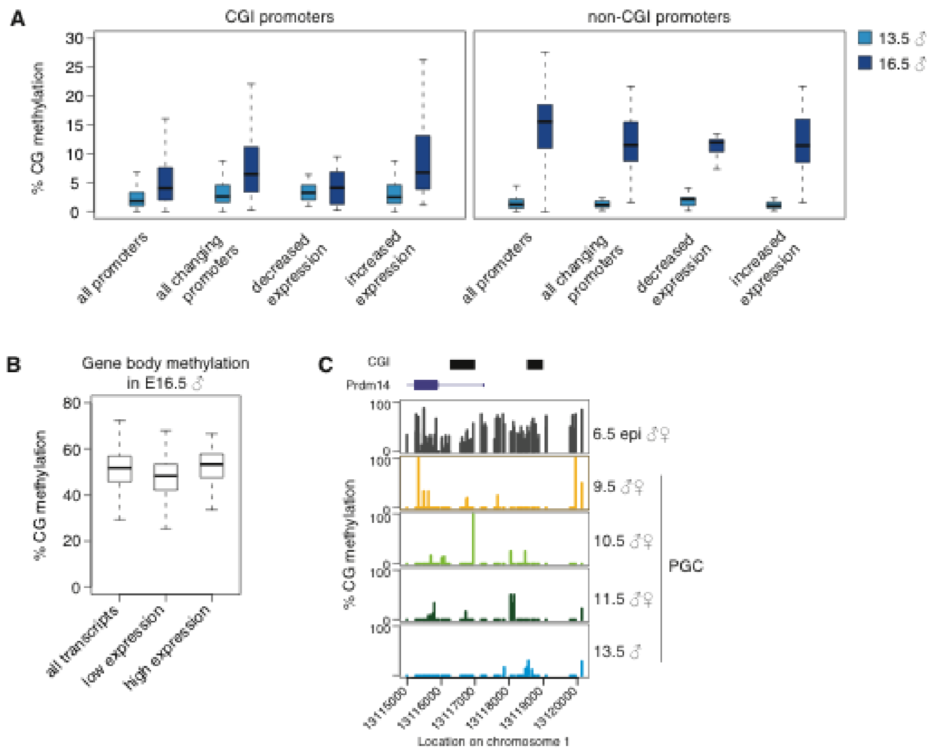
A
CGI promoters
non-CGI promoters
13.5 ♂
16.5 ♂
% CG methylation
30
25
20
15
10
5
0
all promoters
all changing promoters
decreased expression
increased expression
B
Gene body methylation in E16.5 ♂
% CG methylation
80
60
40
20
0
all transcripts
low expression
high expression
C
CGI
Prdm14
6.5 epi ♂♀
9.5 ♂♀
10.5 ♂♀
11.5 ♂♀
13.5 ♂
PGC
% CG methylation
100
0
13115000
13116000
13117000
13118000
13119000
13120000
Location on chromosome 1

Figure S1. Global methylation profiling

(A) Conversion efficiencies in BS-Seq datasets. Measured were levels of CHH methylation with 1kb tiling probes across the genome and shown is the proportion of all probes with varying CHH methylation levels. Note that the largest proportion for each dataset shows complete conversion indicating that conversion efficiencies are high. Two independent biological replicates (1 and 2) are shown for the embryonic samples and two technical replicates of the same BS-Seq library is shown for the J1 samples (highlighted by the asterisk). It is indicated whether the libraries were sequenced on the GIIAx or HiSeq platform.

(B) Global methylation dynamics. Methylation levels of 5kb tiling probes were compared pair wise between two data points (indicated below) and shown is the number of probes with a 25% and 50% increase (top) or decrease (bottom) in methylation levels. Note that the biggest decrease in methylation levels occurs from E6.5 epiblast to E9.5 PGCs and that virtually no *de novo* methylation is observed outside of the comparison between E13.5 and E16.5 male PGCs.

(C) Example figures for the maternally methylated *Kcnq1ot1* DMR (left), the paternally methylated *Rasgrf1* DMR (middle), and the *Xist* promoter region (right). Each bar represents a single CG dinucleotide.

Figure S2. Promoters with slow demethylating kinetics

(A) Methylation levels for the entire genome (left) and across the X chromosome (right) assessed by 5kb tiling probes. Note that the demethylation kinetics for the X chromosome closely resembles those of the whole genome. Outliers are not shown.

(B) Methylation levels for CGIs across the whole genome.

(C) Example figure for a CGI-containing promoter with late demethylation kinetics. Methylation levels seem to be retained especially around the CGI within the promoter. Each bar represents a single CG dinucleotide.

(D) Percentage of promoters that overlap or are nearby (<1kb distance) to confirmed Zfp57-binding sites (Quenneville et al., 2011). Note that there is an enrichment for Zfp57 binding sites in late demethylating CGI promoters.

Figure S3. Resistance to demethylation

(A) Methylation levels for IAPs (left), LINEs (middle), and SINEs (right) across the genome. Note that IAPs retain high levels of methylation whilst LINEs and SINEs undergo significant methylation reprogramming.

(B) Numbers for non-CGI promoters and CGIs that were selected for > 25% methylation at E13.5 in male (left) and female (middle) PGCs. The number of elements overlapping between the lists for male and female PGCs is also indicated (right). Note that resistant non-CGI promoters are more frequent than resistant CGIs and that the overlap between male and female PGCs increases in the presence of an IAP nearby.

(C) Methylation levels of non-CGI promoters that were selected with >25% methylation in male (left) and female (right) PGCs of E13.5 without the presence of an IAP (top) or with an IAP nearby (bottom). Note that non-CGI promoters show consistently higher methylation levels in the presence of an IAP nearby. Outliers are not shown.

Figure S4. Local effect of IAPs on methylation resistance

(A) IAP frequencies found near (< 2kb) or overlapping with CGI-containing or non-CGI promoters. Note that IAPs are rarely found near CGI-containing promoters and frequencies increase drastically for non-CGI promoters selected for > 25% methylation in E13.5 PGCs.

(B) Distance effect of IAPs. Shown are methylation levels for all non-CGI promoters overlapping with or nearby (1kb – 10kb) an IAP in all datasets. Note that the resistance effect is highest up to 2kb away from the IAP.

Figure S5. Resistant CGIs in various datasets.

(A) Methylation levels for CGIs in sperm and oocyte RRBS datasets (Smallwood et al., 2011) selected for > 25% methylation in male (left) and female (right) PGCs of E13.5 without the presence of an IAP (top) or with an IAP nearby (bottom).

(B) VECs were identified as CGIs with > 25% methylation in either male (left) or female (right) PGCs of E13.5 without the presence of an IAP nearby. Each datapoint represents methylation levels for one CGI. Datasets included in this analysis were ES cells (1), blastocyst, sperm, and oocyte (Kobayashi et al., 2012), ES cells (2) (Stadler et al., 2011), 2-cell embryo and ICM (Smith et al., 2012).

Figure S6. Continued hairpin phasing analysis of the LINE1Tf consensus sequence.

Analysis of PGC samples of various time points. Note that in all datasets, there is a strong strand bias for meCGs toward either top or bottom strand highly similar to the outcome for the simulation of passive DNA demethylation (see Figure 5). The number of meCGs is drastically reduced to E13.5 but the strand bias for meCG is preserved throughout the time course.

Figure S7. Continued analysis of passive demethylation pathways.

(A) Expression analysis of the DNA methylation machinery, see Figure 5 for more detail. Note that the expression of the *de novo* methylation machinery is downreglated in early PGCs until E16.5, at which point *Dnmt3a* and *Dnmt3L* seem to increase in expression. *Tet1* is highly expressed in early PGCs, similar to ES cells, and becomes trancriptionally downregulated toward E16.5.

(B) Staining for Np95 and Dnmt1 in replicating PGCs of E13.5 male embryos. Gonads were cultured for 1 hour in the presence of EdU. Note that Np95 localizes predominantly in the cytoplasm of replicating PGCs whilst Dnmt1 signal is strongest in the nucleus. This suggests that whilst the staining pattern for Dnmt1 is in line with the cell cycle stage, the Np95 pattern appears to be cell cycle independent.

(C) Control staining for the Np95 antibody in Np95 KO ES cells. Signal for Np95 staining is virtually absent and this shows high specificity of the antibody.

(D) Sub-cellular localization of Np95 in PGCs throughout development. During the time course analyzed in this study, we found Np95 consistently localized preferentially to the cytoplasm. This is the case for both replicating PGCs (E10.5 – E13.5) and cell cycle arrested PGCs (E13.5 – E16.5). This suggests that uncoupling of the Np95 pattern from the cell cycle may be a common phenomenon throughout PGC development.

Figure S8. Correlation analysis for transcription and DNA methylation.

(A) Methylation levels for CGI-containing (left) and non-CGI (right) promoters of transcripts that change in expression levels from E13.5 to E16.5 in male PGCs. Note that all promoters show increased methylation levels in E16.5 male PGCs and that only CGI-containing promoters of transcripts with decreased expression show a slightly lower increase in methylation levels than all other promoters.

(B) Methylation levels of gene bodies for transcripts that are expressed at low or high levels in E16.5 male PGCs. Only transcripts with a length >25kb were selected and transcripts within the bottom and top 5% of the RPKM were selected for low and high expression, respectively. For each gene with multiple transcripts in this set, the transcript with the highest expression level was selected. Note that gene bodies of transcripts with low expression at E16.5 in male PGCs show lower methylation levels than all other transcripts.

(C) Methylation example graphs for genes from the pluripotency cluster. Each bar represents a single CG dinucleotide.

Supplemental Experimental Procedures

DNA/RNA purification

Genomic DNA and total RNA was extracted either in combination using the Qiagen All Prep DNA/RNA Micro kit according to the manufacturers' instructions or if DNA and RNA were collected separately, Qiagen's QIAmp kit was used for extracting DNA and Life Sciences's Picopure kit for extraction of total RNA according to the manufacturers' instructions.

Annotations

CGI annotations were used based on pull down experiments (Illingworth et al., 2010). Promoters were defined as the region -1kb to +100bp of the transcription start site as annotated in NCBIM37. DMR coordinates were used from E12.5 embryos (Tomizawa et al., 2011). Consensus sequences were used for the analysis of LINE1Tf (DeBerardinis and Kazazian, 1999), LINE1A (Schichman et al., 1993), and IAP1 and IAP2 (Qin et al., 2010). Repeat annotations were extracted from the UCSC RepeatMasker track (mm9 build).

BS-Seq Analysis

Raw sequence reads were trimmed to remove both poor quality calls and adapters using TrimGalore (www.bioinformatics.babraham.ac.uk/projects/trim_galore/). Remaining sequences were mapped to the mouse NCBIM37 genome using Bismark (Krueger and Andrews, 2011), and CG methylation calls were extracted which excluded any duplicate calls from overlapping read ends of short inserts. Read numbers varied greatly depending on the sequencing platform used. For consistency, only the sample with the highest read number for each time point was used in the subsequent analysis. Methylation over a region was calculated for each CG in the region and then these individual values were averaged to give a representative value for the region. Only regions where at least 5 CGs had been measured were taken forward for subsequent analysis. Regions that were covered by a disproportionally high read number and were most likely mapping artifacts were excluded from subsequent analysis. For consensus repeat methylation analysis Bismark was used to map all reads

against a consensus sequence for each repeat class and the methylation calls from these results were analysed directly.

RNA-Seq Analysis

RNA-Seq data was mapped to the mouse NCBIM37 genome assembly using TopHat in conjunction with gene models from Ensembl release 61. Initial quantitation was made by counting the number of reads per transcript corrected per million reads (RPKM). This was adjusted by globally matching the count distributions at the 75th percentile, and then adjusting counts to have a uniform distribution across all samples. For comparisons of absolute expression the quantitated value was corrected by the length of the transcript in kilobases. Differential expression was called by selecting transcripts which changed with a significance of $p<0.05$ after Benjamini and Hochberg correction using a null model constructed from the 1% of transcripts showing the closest average level of observation to estimate experimental noise. Expression clusters were defined by performing hierarchical clustering of transcripts based on a Pearson's correlation across all samples, and selecting groups which were related $R>0.7$ and which contained at least 10 transcripts. Alignments of RNA-Seq data sets against repeat consensus sequences were carried out using Bowtie (v0.12.8, default options) whereby the fraction of aligning reads was scored. Publically available datasets for ESC and MLF (Guttman et al., 2010), J1 (Ficz et al., 2011), ES and EB (Cloonan et al., 2008), and TKO ES cells (Karimi et al., 2011) were included in this analysis where indicated.

Functional Enrichment Analysis (GO analysis)

Function enrichment was analysed by generating a non-redundant list of genes from an initial transcript list and analysing this with the DAVID web tool, using a background of all mouse genes. Groups with a significance of $p<0.05$ after correction were taken to be significant.

Microarray data analysis

The microarray data from early PGCs (Kurimoto et al., 2008) and ES cells (Vincent et al., 2011) was analysed with R/Bioconductor using the package affy. The PGC expression data and the ES data were normalised together using robust multi-array average (RMA) expression measure. Normalised expression data for specific genes was extracted. Where genes had multiple probes, the probe with the highest average (mean) expression was selected as it was reported that this leads to best between-study consistency (Miller et al., 2011).

Supplemental References

Cloonan, N., Forrest, A.R.R., Kolle, G., Gardiner, B.B.A., Faulkner, G.J., Brown, M.K., Taylor, D.F., Steptoe, A.L., Wani, S., Bethel, G., et al. (2008). Stem cell transcriptome profiling via massive-scale mRNA sequencing. Nat Meth *5*, 613–619.

DeBerardinis, R.J., and Kazazian, H.H. (1999). Analysis of the promoter from an expanding mouse retrotransposon subfamily. Genomics *56*, 317–323.

Ficz, G., Branco, M.R., Seisenberger, S., Santos, F., Krueger, F., Hore, T.A., Marques, C.J., Andrews, S., and Reik, W. (2011). Dynamic regulation of 5-hydroxymethylcytosine in mouse ES cells and during differentiation. Nature *473*, 398–402.

Guttman, M., Garber, M., Levin, J.Z., Donaghey, J., Robinson, J., Adiconis, X., Fan, L., Koziol, M.J., Gnirke, A., Nusbaum, C., et al. (2010). Ab initio reconstruction of cell type–specific transcriptomes in mouse reveals the conserved multi-exonic structure of lincRNAs. Nat Biotechnol *28*, 503–510.

Illingworth, R.S., Gruenewald-Schneider, U., Webb, S., Kerr, A.R.W., James, K.D., Turner, D.J., Smith, C., Harrison, D.J., Andrews, R., and Bird, A.P. (2010). Orphan CpG islands identify numerous conserved promoters in the mammalian genome. PLoS Genet *6*, e1001134

Karimi, M.M., Goyal, P., Maksakova, I.A., Bilenky, M., Leung, D., Tang, J.X., Shinkai, Y., Mager, D.L., Jones, S., Hirst, M., et al. (2011). DNA Methylation and SETDB1/H3K9me3 Regulate Predominantly Distinct Sets of Genes, Retroelements, and Chimeric Transcripts in mESCs. Stem Cell *8*, 676–687.

Kobayashi, H., Sakurai, T., Imai, M., Takahashi, N., Fukuda, A., Yayoi, O., Sato, S., Nakabayashi, K., Hata, K., Sotomaru, Y., et al. (2012). Contribution of Intragenic DNA Methylation in Mouse Gametic DNA Methylomes to Establish Oocyte-Specific Heritable Marks. PLoS Genet *8*, e1002440.

Krueger, F., and Andrews, S.R. (2011). Bismark: a flexible aligner and methylation caller for Bisulfite-Seq applications. Bioinformatics *27*, 1571–1572.

Kurimoto, K., Yabuta, Y., Ohinata, Y., Shigeta, M., Yamanaka, K., and Saitou, M. (2008). Complex genome-wide transcription dynamics orchestrated by Blimp1 for the specification of the germ cell lineage in mice. Genes Dev *22*, 1617–1635.

Miller, J.A., Cai, C., Langfelder, P., Geschwind, D.H., Kurian, S.M., Salomon, D.R., and Horvath, S. (2011). Strategies for aggregating gene expression data: The collapseRows R function. BMC Bioinformatics *12*, 322.

Qin, C., Wang, Z., Shang, J., Bekkari, K., Liu, R., Pacchione, S., McNulty, K.A., Ng, A., Barnum, J.E., and Storer, R.D. (2010). Intracisternal A particle genes: Distribution in the mouse genome, active subtypes, and potential roles as species-specific mediators of susceptibility to cancer. Mol. Carcinog. *49*, 54–67.

Quenneville, S., Verde, G., Corsinotti, A., Kapopoulou, A., Jakobsson, J., Offner, S., Baglivo, I., Pedone, P.V., Grimaldi, G., Riccio, A., et al. (2011). In Embryonic Stem Cells, ZFP57/KAP1 Recognize a Methylated Hexanucleotide to Affect Chromatin and DNA Methylation of Imprinting Control Regions. Mol Cell *44*, 361–372.

Schichman, S.A., Adey, N.B., Edgell, M.H., and Hutchison, C.A. (1993). L1 A-monomer tandem arrays have expanded during the course of mouse L1 evolution. Mol. Biol. Evol. *10*,

552–570.

Smallwood, S.A., Tomizawa, S.-I., Krueger, F., Ruf, N., Carli, N., Segonds-Pichon, A., Sato, S., Hata, K., Andrews, S.R., and Kelsey, G. (2011). Dynamic CpG island methylation landscape in oocytes and preimplantation embryos. Nat Genet *43*, 811–814.

Smith, Z.D., Chan, M.M., Mikkelsen, T.S., Gu, H., Gnirke, A., Regev, A., and Meissner, A. (2012). A unique regulatory phase of DNA methylation in the early mammalian embryo. Nature *484*, 339–344.

Stadler, M.B., Murr, R., Burger, L., Ivanek, R., Lienert, F., Schöler, A., van Nimwegen, E., Wirbelauer, C., Oakeley, E.J., Gaidatzis, D., et al. (2011). DNA-binding factors shape the mouse methylome at distal regulatory regions. Nature *480*, 490–495.

Tomizawa, S.I., Kobayashi, H., Watanabe, T., Andrews, S., Hata, K., Kelsey, G., and Sasaki, H. (2011). Dynamic stage-specific changes in imprinted differentially methylated regions during early mammalian development and prevalence of non-CpG methylation in oocytes. Development *138*, 811–820.

Vincent, J.J., Li, Z., Lee, S.A., Liu, X., Etter, M.O., Diaz-Perez, S.V., Taylor, S.K., Gkountela, S., Lindgren, A.G., and Clark, A.T. (2011). Single Cell Analysis Facilitates Staging of Blimp1-Dependent Primordial Germ Cells Derived from Mouse Embryonic Stem Cells. PLoS ONE *6*, e28960.
